# Effectiveness of Interventions for Dysphagia in Parkinson Disease: A Systematic Review

**DOI:** 10.1044/2021_AJSLP-21-00145

**Published:** 2021-12-10

**Authors:** Pooja Gandhi, Catriona M. Steele

**Affiliations:** a Swallowing Rehabilitation Research Laboratory, Toronto Rehabilitation Institute—University Health Network, Ontario, Canada; b Rehabilitation Sciences Institute, Faculty of Medicine, University of Toronto, Ontario, Canada

## Abstract

**Purpose:**

Dysphagia is a common sequela of Parkinson disease (PD) and is associated with malnutrition, aspiration pneumonia, and mortality. This review article synthesized evidence regarding the effectiveness of interventions for dysphagia in PD.

**Method:**

Electronic searches were conducted in Ovid MEDLINE, Embase, Cochrane Central Register of Controlled Trials, CINAHL, and speechBITE. Of the 2,015 articles identified, 26 met eligibility criteria: interventional or observational studies with at least five or more participants evaluating dysphagia interventions in adults with PD-related dysphagia, with outcomes measured using videofluoroscopic swallowing study (VFSS), fiberoptic endoscopic evaluation of swallowing (FEES), or electromyography (EMG). Risk of bias (RoB) was evaluated using the Evidence Project tool and predetermined criteria regarding the rigor of swallowing outcome measures.

**Results:**

Interventions were classified as follows: pharmacological (*n* = 11), neurostimulation (*n* = 8), and behavioral (*n* = 7). Primary outcome measures varied across studies, including swallowing timing, safety, and efficiency, and were measured using VFSS (*n* = 17), FEES (*n* = 6), and EMG (*n* = 4). Critical appraisal of study findings for RoB, methodological rigor, and transparency showed the majority of studies failed to adequately describe contrast media used, signal acquisition settings, and rater blinding to time point. Low certainty evidence generally suggested improved swallow timing with exercises with biofeedback and deep brain stimulation (DBS), improved safety with DBS and expiratory muscle strength training, and improved efficiency with the Lee Silverman Voice Treatment and levodopa.

**Conclusions:**

Studies with lower RoB and greater experimental rigor showed potential benefit in improving swallowing efficiency but not safety. Further research investigating discrete changes in swallowing pathophysiology post-intervention is warranted to guide dysphagia management in PD.

**Supplemental Material:**

https://doi.org/10.23641/asha.17132162

Parkinson disease (PD) is one of the most common neurological disorders internationally, with a rising prevalence with age (De Rijk et al., 1995; Pringsheim et al., 2014; von Campenhausen et al., 2005). In the context of increasing life expectancies globally, a steady increase in PD is anticipated, with almost 9 million people affected by 2030 (Dorsey et al., 2007; Pringsheim et al., 2014; Suttrup & Warnecke, 2016). This debilitating condition is known to affect the central and peripheral nervous systems, with the most salient histopathological feature being the presence of α-synuclein aggregates (Lewy bodies and Lewy neurites; Braak et al., 2004; Mu et al., 2013). Disrupted neural signaling in PD is also attributed to neuroinflammatory processes, mitochondrial dysfunction, and altered apoptosis pathways (Rocha et al., 2018). Although PD primarily involves degeneration of the nigrostriatal dopaminergic pathway (Braak et al., 2004; Chaudhuri et al., 2006; Mu et al., 2013; Pringsheim et al., 2014), it also impacts other neural systems, causing neuromediator dysfunctions, which, in turn, result in complex functional deficits (Jellinger, 1991; Mu et al., 2013). Bulbar dysfunctions (including dysphagia, hypophonia, dysarthria, and sialorrhea) are frequently noted in PD and are equally, if not more, debilitating as the hallmark features (Braak et al., 2004; Chaudhuri et al., 2006; Leopold & Kagel, 1997; Miller et al., 2006; Nilsson et al., 1996; Potulska et al., 2003). In particular, dysphagia is significantly associated with malnutrition and aspiration pneumonia in PD, with the latter being a leading cause of death in this population (Baijens & Speyer, 2009; Beyer et al., 2001; Johnston et al., 1995; Kalf et al., 2012; Wang et al., 2002). Dysphagia also negatively impacts quality of life, with patients reporting restricted participation in social activities involving eating and drinking (Andersson & Sidenvall, 2001; Clarke et al., 1998; Ekberg et al., 2002; Farri et al., 2007; Plowman-Prine et al., 2009).

There is limited evidence regarding the pathophysiological mechanisms underlying oropharyngeal dysphagia in PD. Treatments frequently involve a combination of rehabilitative and compensatory approaches (Smith et al., 2012). Rehabilitative approaches include resistance exercises for the laryngeal, respiratory, and orofacial muscles. Compensatory strategies aim to make eating and drinking safer without inducing longer lasting changes in swallowing physiology.

The majority of previously published systematic reviews examining the relative effectiveness of dysphagia treatments date back to 2014 or earlier (Baijens & Speyer, 2009; Deane et al., 2001; Smith et al., 2012; Van Hooren et al., 2014) and lack comprehensive consideration of different treatment modalities (i.e., pharmacological, neurostimulation, and behavioral approaches). Additionally, these historical reviews, together with a more recent review by López-Liria et al. (2020), display unevenness in the appraisal of study quality, rigor, and transparency. Thus, the purpose of this systematic review was to identify and evaluate literature regarding the efficacy of pharmacological, neurostimulation, and behavioral interventions as distinct categories for the treatment of dysphagia in patients with PD as well as to carefully scrutinize and critically appraise study findings, methodological rigor, and transparency in order to guide evidence-informed clinical decision-making.

With respect to experimental rigor and transparency, we were particularly interested to review details regarding the instrumental methods that were used to measure treatment outcomes. Videofluoroscopic swallowing study (VFSS) and fiberoptic endoscopic evaluation of swallowing (FEES) are widely accepted as gold standard approaches for dysphagia diagnosis in clinical practice. However, even these procedures have been criticized for a lack of standards and poor interrater agreement (Kuhlemeier et al., 1998; McCullough et al., 2001; Ott, 1998; Plowman & Humbert, 2018; Swan et al., 2019; Tohara et al., 2010). Several recent papers note that nonstandardized VFSS practices persist in clinical practice, both in the United States and internationally (Boaden et al., 2020, 2021; Martin-Harris et al., 2021). Accordingly, we felt it was important to appraise the rigor with which the methods of these instrumental examinations were performed and reported in research studies measuring treatment outcomes for dysphagia in PD. Variations that may impact diagnostic accuracy and measures of swallowing physiology include, but are not limited to, variations in signal acquisition settings and frame rate (e.g., Bonilha et al., 2013; Peladeau-Pigeon & Steele, 2013, 2015), contrast media concentration (e.g., Steele et al., 2013), the consistencies studied (e.g., Steele, Peladeau-Pigeon, et al., 2019), bolus volume (e.g., Butler et al., 2011), and whether or not participants were instructed to wait for a cue before initiating a swallow (e.g., Daniels et al., 2007; Nagy et al., 2013). It is important to understand not only the protocols that were used but also how the data were processed. For example, in a protocol containing several sips of thin liquid barium, it is critical to know whether the resulting data represent the mean value across task repetitions within participants, reflect data for all swallows (with appropriate handling of repeated measures), or reflect data for a particular swallow (e.g., the first bolus or the bolus showing the worst score on a particular parameter). Furthermore, given that studies suggest that penetration–aspiration and swallowing physiology may vary within an individual across repeated sips of thin liquid (Steele, Mukherjee, et al., 2019) or across tasks of different consistencies and volumes (Hazelwood et al., 2017), an important aspect of rigor in reporting is to understand the number of boluses of each consistency and volume that were included in a protocol. For this purpose, we developed an a priori criterion-based set of 10 quality indicators based on questions proposed for the assessment of study quality and rigor in two recent reviews exploring dysphagia treatment outcomes (Bahia & Lowell, 2020; Mancopes et al., 2020). As listed in Table 1, these included questions regarding the number of boluses and consistencies tested, bolus volumes, contrast media, recording settings, the time point of rating, rater blinding, and reliability.

**Table 1.** Questions used in the appraisal of rigor in instrumental evaluations of swallowing.

Was more than one bolus tested?
Was more than one consistency tested?
Were details regarding volume reported?
If used, were details regarding barium (or other contrast) concentration reported?
Were details regarding recording settings reported (specifically signal acquisition rate)?
Were ratings made post hoc from recorded signals (as opposed to online)?
Were raters blinded to participant ID/group assignment?
Were raters blinded to time point/condition?
Were interrater reliability statistics reported?
Were intrarater reliability statistics reported?

## Method

### Literature Search

A comprehensive literature search was carried out by a trained health information specialist in May 2019. The search was conducted according to the *Cochrane Handbook for Systematic Reviews of Interventions* (Higgins et al., 2019) and the Preferred Reporting Items for Systematic Reviews and Meta-Analyses (PRISMA) statement (Moher et al., 2009). Electronic database searches were conducted in Ovid MEDLINE, Embase, CINAHL, speechBITE, and Cochrane Central Register of Controlled Trials, with keywords and subject headings related to swallowing, dysphagia, and PD. The full search strategy can be found in the Appendix. The search was limited to peer-reviewed English-language human studies published from database inception to May 2019. Reference lists of all articles included for synthesis were hand-searched for additional relevant articles.

### Selection Criteria

Studies were eligible if they included adult (over 18 years of age) patients with idiopathic PD and associated oropharyngeal dysphagia as well as examined the effect of a dysphagia-targeted intervention with pre- and posttreatment comparison. Studies describing individuals with non-idiopathic parkinsonian syndromes were excluded. Studies were required to report outcomes using one or more of the following instrumental methods: VFSS, FEES, and/or electromyography (EMG). Studies were excluded if they did not report primary data (i.e., editorials, systematic reviews, book chapters), were single-case reports, or were limited to interventions for esophageal dysphagia with no oropharyngeal component. Conference proceedings and other gray literature were also excluded. Two reviewers independently screened the titles and abstracts of identified citations, followed by a full-text review of potentially eligible studies. Disagreements regarding inclusion were resolved by consensus.

### Data Extraction and Quality Appraisal

Two reviewers performed data extraction independently and in duplicate using data extraction forms. The information extracted included study characteristics; patient demographics; characterization of PD based on severity and duration; intervention type, intensity, and duration; and reported swallowing outcomes. Risk of bias (RoB) was evaluated according to a tool developed by the Evidence Project (Kennedy et al., 2019), which has been validated across both randomized and nonrandomized studies. This tool includes eight criteria, each of which is rated as being present (yes) or not (no), not reported (em dash), or not applicable (blank cell). The tool assesses whether (a) a cohort of participants was followed over time and included multiple assessments with the same participants, (b) intervention outcomes were compared against a control or comparison group, (c) pre- and post-intervention data were reported, (d) there was random assignment of participants to the intervention, (e) participants were randomly selected for enrollment from an available pool of candidates, (f) the study group had a follow-up rate of 80% or more, (g) the comparison groups were equivalent on sociodemographic factors, and (h) comparison groups were equivalent at baseline on the selected outcome measures. Overall RoB was classified as high if more than 80% of the criteria were scored as absent or not reported and low if at least 80% or more of the criteria were rated as being present. In cases where particular criteria were not applicable, the denominator was adjusted to reflect the number of articles for which the criterion applied. The rigor and reporting transparency of the instrumental methods were appraised using the criteria in Table 1, including questions regarding the number of boluses and consistencies tested, bolus volumes, contrast media, recording settings, the time point of rating, rater blinding, and reliability.

### Data Synthesis

Where sufficient data were available, a meta-analysis was planned. Given heterogeneity in study designs and a paucity of “poolable” data for the outcomes of interest, the method and results were summarized descriptively for all reported videofluoroscopic, endoscopic, and electromyographic measures, and overall findings were summarized narratively.

## Results

**Figure 1.**
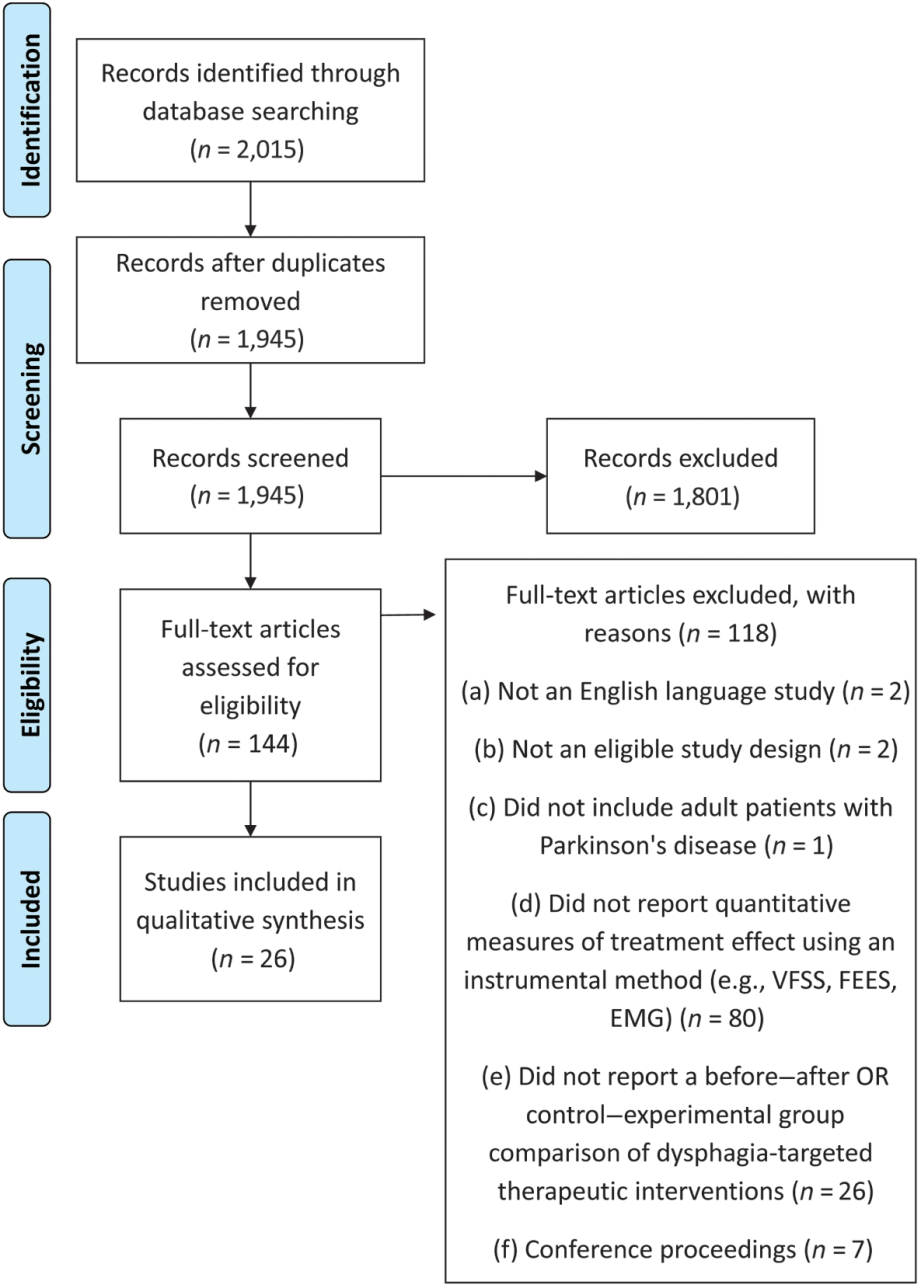
Preferred Reporting Items for Systematic Reviews and Meta-Analyses diagram showing the process followed for selecting articles for inclusion in the review. VFSS = videofluoroscopic swallowing study; FEES = fiberoptic endoscopic evaluation of swallowing; EMG = electromyography.

Figure 1 shows the PRISMA diagram summarizing the search strategy and results for this review article. Of the 2,015 citations identified by the search, 1,945 were screened for eligibility after duplicates were removed. Of these, 144 studies were considered potentially eligible, requiring full-text review, and 26 were found to meet all criteria for inclusion and synthesis (Alfonsi et al., 2017; Argolo et al., 2013; Athukorala et al., 2014; Baijens et al., 2013; Bushmann et al., 1989; Ciucci et al., 2008; El Sharkawi et al., 2002; Fuh et al., 1997; Hirano et al., 2015; Hunter et al., 1997; Khedr et al., 2019; Kondo et al., 2017; Kulneff et al., 2013; Lengerer et al., 2012; Michou et al., 2014; Miles et al., 2017; Monte et al., 2005; Pitts et al., 2009; Stegemöller et al., 2017; Sundstedt, Holmén, et al., 2017; Sundstedt et al., 2012; Tawadros et al., 2012; Tison et al., 1996; Troche et al., 2010; Warnecke et al., 2016; Xie et al., 2015). Interrater agreement between two reviewers was calculated, using the kappa statistic, to be .59 (moderate agreement) at the title and abstract screening stage and .71 (substantial agreement) at the full-text review stage (McHugh, 2012).

### Study Characteristics

A summary of study characteristics and participant demographics for the included studies can be found in Supplemental Material S1. The majority of studies were interventional. Eleven studies were randomized controlled trials (RCTs). Nine studies were before–after trials. Four studies were prospective cohort studies, and two studies were retrospective cohort studies. All studies were single-center studies and were conducted across 12 countries, including Australia, Brazil, France, Germany, Italy, Japan, the Netherlands, New Zealand, Sweden, Taiwan, the United Kingdom, and the United States of America.

Sample sizes varied widely, ranging from six to 90 participants, with a mean age ranging from 49.3 to 75.0 years. Of the 24 studies reporting descriptive statistics regarding the age of participants with PD, 21 reported a mean or median age of over 60 years for the patient participants (Alfonsi et al., 2017; Athukorala et al., 2014; Bushmann et al., 1989; Ciucci et al., 2008; El Sharkawi et al., 2002; Fuh et al., 1997; Hirano et al., 2015; Hunter et al., 1997; Khedr et al., 2019; Kulneff et al., 2013; Michou et al., 2014; Miles et al., 2017; Monte et al., 2005; Pitts et al., 2009; Stegemöller et al., 2017; Sundstedt et al., 2012; Tawadros et al., 2012; Tison et al., 1996; Troche et al., 2010; Warnecke et al., 2016; Xie et al., 2015). The remaining three studies had participants with ages between 45 and 60 years (Argolo et al., 2013; Lengerer et al., 2012; Sundstedt, Holmén, et al., 2017). Across the 21 studies reporting the gender distribution of included participants, the mean proportion of male participants was 70% (Alfonsi et al., 2017; Argolo et al., 2013; Athukorala et al., 2014; Baijens et al., 2013; Bushmann et al., 1989; Ciucci et al., 2008; El Sharkawi et al., 2002; Hirano et al., 2015; Hunter et al., 1997; Kondo et al., 2017; Kulneff et al., 2013; Lengerer et al., 2012; Michou et al., 2014; Miles et al., 2017; Monte et al., 2005; Pitts et al., 2009; Stegemöller et al., 2017; Sundstedt, Holmén, et al., 2017; Sundstedt et al., 2012; Troche et al., 2010; Xie et al., 2015).

Of the 13 studies reporting the criteria used to confirm the diagnosis of PD, 10 used the United Kingdom Parkinson's Disease Society Brain Bank Clinical Diagnostic Criteria (Alfonsi et al., 2017; Argolo et al., 2013; Hirano et al., 2015; Hunter et al., 1997; Khedr et al., 2019; Lengerer et al., 2012; Michou et al., 2014; Pitts et al., 2009; Troche et al., 2010; Warnecke et al., 2016). Of the remaining three studies, one based eligibility on a neurologist-confirmed diagnosis (Athukorala et al., 2014), one required participants to have at least two symptoms from a set of three (resting tremor, rigidity, and/or bradykinesia; Fuh et al., 1997), and one determined eligibility using criteria outlined in a textbook (Tison et al., 1996). Of the 19 studies reporting the severity of PD, 16 used the Hoehn and Yahr scale (Argolo et al., 2013; Athukorala et al., 2014; Baijens et al., 2013; Bushmann et al., 1989; Ciucci et al., 2008; El Sharkawi et al., 2002; Fuh et al., 1997; Hirano et al., 2015; Hoehn & Yahr, 1967; Khedr et al., 2019; Michou et al., 2014; Monte et al., 2005; Pitts et al., 2009; Sundstedt, Holmén, et al., 2017; Tison et al., 1996; Troche et al., 2010; Warnecke et al., 2016). The remaining three studies (Lengerer et al., 2012; Sundstedt et al., 2012; Xie et al., 2015) used the Unified Parkinson's Disease Rating Scale, Part III (Martinez-Martin et al., 1994).

The majority of studies (*n* = 17) measured outcomes using VFSS (Argolo et al., 2013; Baijens et al., 2013; Bushmann et al., 1989; Ciucci et al., 2008; El Sharkawi et al., 2002; Fuh et al., 1997; Hirano et al., 2015; Hunter et al., 1997; Khedr et al., 2019; Lengerer et al., 2012; Michou et al., 2014; Miles et al., 2017; Monte et al., 2005; Pitts et al., 2009; Tison et al., 1996; Troche et al., 2010; Xie et al., 2015). FEES was used to measure swallowing outcomes in six studies (Baijens et al., 2013; Kondo et al., 2017; Kulneff et al., 2013; Sundstedt, Holmén, et al., 2017; Sundstedt et al., 2012; Warnecke et al., 2016). The four remaining studies reported outcomes measured using EMG, of which three used surface EMG (sEMG; Athukorala et al., 2014; Stegemöller et al., 2017; Tawadros et al., 2012) and one used intramuscular EMG (Alfonsi et al., 2017).

### Reported Results

Supplemental Material S1 also summarizes the intervention approaches used and the reported results for the included studies. These will be briefly described by intervention type.

#### Pharmacological Interventions

Across the 26 studies included for synthesis, nine explored the effects of dopamine agonist medications on swallowing (e.g., levodopa, carbidopa, apomorphine, domperidone, rotigotine). Bushmann et al. (1989) found that administering levodopa with carbidopa led to partial improvements in swallowing efficiency in the form of faster transit times and reduced residue. Mixed results were reported by Fuh et al. (1997), with improvements seen in six of 12 patients receiving levodopa with benserazide, including reductions in pharyngeal residue. This was concordant with results from the work of Warnecke et al. (2016), who demonstrated improvement in swallowing efficiency and residue with levodopa administration in seven of 15 patients. By contrast, Monte et al. (2005) observed no improvements in swallowing efficiency with levodopa. Similarly, Tawadros et al. (2012) found no influence of levodopa on submental sEMG burst parameters, laryngeal parameters, or number of swallows at any volume.

Two studies reported shorter oral preparatory phase durations and shorter pharyngeal transit times after the administration of apomorphine (Hunter et al., 1997; Tison et al., 1996). The Tison et al. (1996) study also reported reductions in residue and piecemeal swallowing in seven of eight patients and improvements in airway protection in two of three patients with laryngeal penetration at baseline. Hirano et al. (2015) investigated the effectiveness of a rotigotine patch in improving swallowing efficiency and reported shorter pharyngeal transit times in all six patients.

Other pharmacological interventional studies included a single study that explored the effect of botulinum toxin injections on opening of the upper esophageal sphincter (UES) in a mixed sample of patients with neurological diagnoses, including 12 with PD (Alfonsi et al., 2017). Among these 12 patients, six were reported to show a strong response after a first injection, with four more showing partial response. Finally, Kondo et al. (2017) explored the effects of applying capsaicin ointment to the external auditory canal, with the goal of stimulating the vagus nerve. The experimental group (*n* = 10) included one participant with PD. They reported groupwise improvements in glottal closure, timing, and efficiency in the experimental group compared with no changes in the placebo group, which included two participants with PD.

#### Neurostimulation Interventions

Several studies explored the impact of neurostimulation approaches to intervention, with six studying the impact of deep brain stimulation (DBS). Ciucci et al. (2008) reported improvements in swallowing timing and pharyngeal composite score. Lengerer et al. (2012) also reported improvements in swallowing timing and latency. Xie et al. (2015) reported that 60-Hz stimulation reduced the frequency of aspiration by 57%, with benefits persisting at a 6-week follow-up assessment. On the contrary, Sundstedt et al. (2012) found that initial reductions of premature spillage were not maintained 1 year post. The same group replicated these results in 2017, noting no changes in premature spillage, penetration–aspiration, or pharyngeal residue (Sundstedt, Holmén, et al., 2017). Similarly, Kulneff et al. (2013) found no significant effect of DBS on FEES parameters, including secretions, premature spillage, penetration–aspiration, and residue. Within this category, a single study described the impact of transcutaneous neuromuscular electrical stimulation (VitalStim; Baijens et al., 2013), showing no differences for any visuoperceptual measures on FEES or VFSS. Similarly, a single study explored the impact of repetitive transcranial magnetic stimulation (rTMS) on swallowing safety and efficiency (Khedr et al., 2019) but found no differences in penetration–aspiration or residue between sham and real rTMS groups.

#### Behavioral Interventions

The remaining seven studies in this review article explored the effects of behavioral interventions. Of these, two measured the effect of the Lee Silverman Voice Treatment (LSVT) program. El Sharkawi et al. (2002) found the LSVT to be effective in shortening timing measures and reducing oral residue for 3- and 5-ml liquid swallows posttreatment. Miles et al. (2017) showed improvements after the LSVT in the form of reduced pharyngeal residue and significantly increased duration and maximal opening of the UES. Two studies described the effects of expiratory muscle strength training (EMST; Pitts et al., 2009; Troche et al., 2010), with both reporting significant improvements in penetration–aspiration after training. The three remaining studies described exercise-based interventions, as follows:

1. Argolo et al. (2013) employed an exercise program targeting “strength and range of motion of the mouth, larynx and pharyngeal structures, coordination between breathing and swallowing, and airway protection.”
2. Athukorala et al. (2014) used sEMG biofeedback to train skills in generating submental muscle contractions with precise timing and amplitude.
3. Stegemöller et al. (2017) studied the effects of a therapeutic singing intervention.

Neither the Argolo et al. study nor the Stegemöller et al. study observed any improvements in swallowing after the intervention. Athukorala et al. noted some changes in timing measures of submental muscle contraction for dry swallows.

Summaries of the RoB evaluations performed using the Evidence Project tool can be found in Table 2. The study by Baijens et al. (2013) included separate reporting and analysis of outcomes measured using VFSS and FEES and is therefore included twice, reflecting separate appraisals of these two portions of the study. Common concerns with respect to bias included failure to report any information regarding whether participants were randomly selected for assessment (17 of 27 assessments; Alfonsi et al., 2017; Athukorala et al., 2014; Baijens et al., 2013; Bushmann et al., 1989; Ciucci et al., 2008; Fun et al., 1997; Hunter et al., 1997; Kondo et al., 2017; Lengerer et al., 2012; Michou et al., 2014; Pitts et al., 2009; Stegemöller et al., 2017; Tawadros et al., 2012; Tison et al., 1996; Troche et al., 2010; Xie et al., 2015). Of the 10 studies where this item was reported, none used random selection during participant recruitment (Argolo et al., 2013; El Sharkawi et al., 2002; Hirano et al., 2015; Khedr et al., 2019; Kulneff et al., 2013; Miles et al., 2017; Monte et al., 2005; Sundstedt, Holmén, et al., 2017; Sundstedt et al., 2012; Warnecke et al., 2016). Attrition rates were below 20% across all studies, except two (Monte et al., 2005; Warnecke et al., 2016). Overall, a high RoB was identified in 22 assessments (Alfonsi et al., 2017; Argolo et al., 2013; Athukorala et al., 2014; Bushmann et al., 1989; Ciucci et al., 2008; El Sharkawi et al., 2002; Fuh et al., 1997; Hirano et al., 2015; Hunter et al., 1997; Kondo et al., 2017; Kulneff et al., 2013; Lengerer et al., 2012; Miles et al., 2017; Monte et al., 2005; Pitts et al., 2009; Stegemöller et al., 2017; Sundstedt, Holmén, et al., 2017; Sundstedt et al., 2012; Tawadros et al., 2012; Tison et al., 1996; Warnecke et al., 2016; Xie et al., 2015).

**Table 2.** Risk-of-bias evaluation using the Evidence Project tool.

Study	Cohort study?	Control/ comparison group?	Pre- and post-intervention data reported?	Random assignment of participants to intervention?	Random selection of participants for enrollment?	Follow-up rate of 80% or more?	Comparison groups equivalent on sociodemographics?	Comparison groups equivalent at baseline on disclosure?	Overall risk-of-bias score
Alfonsi et al. (2017)	Yes	No	No	No	—	Yes	No	No	2/8 = 25.0%
Argolo et al. (2013)	Yes	No	Yes		No	Yes			2/5 = 40.0%
Athukorala et al. (2014)	Yes	No	Yes		—	Yes			2/5 = 40.0%
Baijens et al. (2013): VFSS arm	Yes	Yes	Yes	Yes	—	Yes	Yes	Yes	7/8 = 87.5%
Baijens et al. (2013): FEES arm	Yes	Yes	Yes	Yes	—	Yes	Yes	Yes	7/8 = 87.5%
Bushmann et al. (1989)	Yes	Yes	Yes		—	Yes	Yes	No	5/7 = 71.4%
Ciucci et al. (2008)	Yes	No	Yes		—	Yes			3/5 = 60.0%
El Sharkawi et al. (2002)	Yes	No	Yes		No	Yes			3/5 = 60.0%
Fuh et al. (1997)	Yes	No	Yes		—	Yes			3/5 = 60.0%
Hirano et al. (2015)	Yes	No	Yes		No	Yes			3/5 = 60.0%
Hunter et al. (1997)	Yes	No	Yes		—	Yes			3/6 = 60.0%
Khedr et al. (2019)	Yes	Yes	Yes	Yes	No	Yes	Yes	Yes	7/8 = 87.5%
Kondo et al. (2017)	No	Yes	Yes	Yes	—	Yes	Yes	Yes	6/8 = 75.0%
Kulneff et al. (2013)	Yes	No	Yes		No	Yes			3/5 = 60.0%
Lengerer et al. (2012)	Yes	No	Yes		—	Yes			3/5 = 60.0%
Michou et al. (2014)	Yes	Yes	Yes		—	Yes	Yes	Yes	6/7 = 85.7%
Miles et al. (2017)	Yes	No	Yes		No	Yes			3/5 = 60.0%
Monte et al. (2005)	Yes	Yes	No	No	No	No	Yes	No	3/8 = 37.5%
Pitts et al. (2009)	Yes	No	Yes		—	Yes			3/5 = 60.0%
Stegemöller et al. (2017)	Yes	Yes	Yes	No	—	Yes	Yes	Yes	6/8 = 75.0%
Sundstedt et al. (2012)	Yes	No	Yes		No	Yes			3/5 = 60.0%
Sundstedt, Holmén, et al. (2017)	Yes	No	Yes		No	Yes			3/5 = 60.0%
Tawadros et al. (2012)	Yes	Yes	Yes		—	Yes	Yes	No	5/7 = 71.4%
Tison et al. (1996)	Yes	No	Yes		—	Yes			3/5 = 60.0%
Troche et al. (2010)	Yes	Yes	Yes	Yes	—	Yes	Yes	Yes	7/8 = 87.5%
Warnecke et al. (2016)	Yes	No	Yes		No	No			2/5 = 40.0%
Xie et al. (2015)	Yes	No	Yes		—	Yes			3/5 = 60.0%

*Note.* Em dashes indicate data not reported. VFSS = videofluoroscopic swallowing study; FEES = fiberoptic endoscopic evaluation of swallowing.

**Figure 2.**
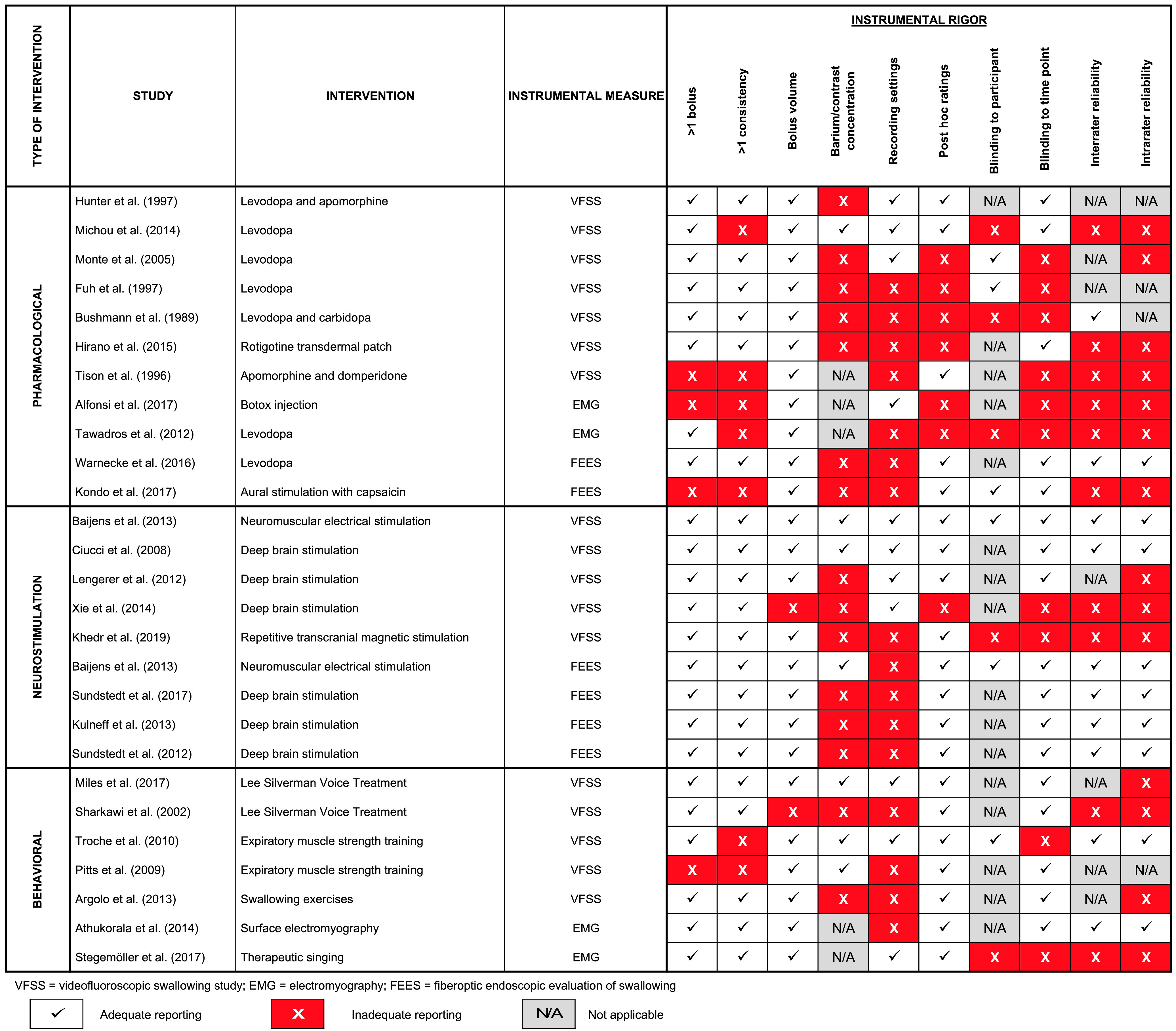
Appraisal of rigor used in instrumental measures of swallowing. Included studies are grouped by intervention type and listed in descending order of instrumental rigor.

Figure 2 shows the results of the appraisal of rigor in the performance and reporting of instrumental measures of swallowing; these results reveal several shortcomings of the selected studies. Four studies reported outcomes based on swallowing of only a single bolus (Alfonsi et al., 2017; Kondo et al., 2017; Pitts et al., 2009; Tison et al., 1996). Seven studies reported results for only a single bolus consistency (Alfonsi et al., 2017; Kondo et al., 2017; Michou et al., 2014; Pitts et al., 2009; Tawadros et al., 2012; Tison et al., 1996; Troche et al., 2010). By contrast, all of the selected studies, with the exception of one (El Sharkawi et al., 2002), reported details regarding the bolus volumes tested. A methodological detail that was inadequately reported in multiple studies (*n* = 15) was the identification of the brands, concentrations, or preparation methods of barium or other contrast agents used (Argolo et al., 2013; Bushmann et al., 1989; El Sharkawi et al., 2002; Fuh et al., 1997; Hirano et al., 2015; Hunter et al., 1997; Khedr et al., 2019; Kondo et al., 2017; Kulneff et al., 2013; Lengerer et al., 2012; Monte et al., 2005; Sundstedt, Holmén, et al., 2017; Sundstedt et al., 2012; Warnecke et al., 2016; Xie et al., 2015). Similarly, details regarding recording settings and signal acquisition rates were missing from 15 studies (Argolo et al., 2013; Athukorala et al., 2014; Bushmann et al., 1989; El Sharkawi et al., 2002; Fuh et al., 1997; Hirano et al., 2015; Khedr et al., 2019; Kondo et al., 2017; Kulneff et al., 2013; Pitts et al., 2009; Sundstedt, Holmén, et al., 2017; Sundstedt et al., 2012; Tawadros et al., 2012; Tison et al., 1996; Warnecke et al., 2016).

In seven studies, rating was described as being performed online as opposed to post hoc from recorded signals (Alfonsi et al., 2017; Bushmann et al., 1989; Fuh et al., 1997; Hirano et al., 2015; Monte et al., 2005; Tawadros et al., 2012; Xie et al., 2015). Rating by multiple individuals was used in 20 of the selected studies (Alfonsi et al., 2017; Athukorala et al., 2014; Baijens et al., 2013; Bushmann et al., 1989; Ciucci et al., 2008; El Sharkawi et al., 2002; Hirano et al., 2015; Khedr et al., 2019; Kondo et al., 2017; Kulneff et al., 2013; Michou et al., 2014; Stegemöller et al., 2017; Sundstedt, Holmén, et al., 2017; Sundstedt et al., 2012; Tawadros et al., 2012; Tison et al., 1996; Troche et al., 2010; Warnecke et al., 2016; Xie et al., 2015). Notably, interrater reliability statistics were not reported in half (i.e., 10) of these studies (Alfonsi et al., 2017; El Sharkawi et al., 2002; Hirano et al., 2015; Khedr et al., 2019; Kondo et al., 2017; Michou et al., 2014; Stegemöller et al., 2017; Tawadros et al., 2012; Tison et al., 1996; Xie et al., 2015). In five studies, raters were not blinded to participant identity or group assignment (Bushmann et al., 1989; Khedr et al., 2019; Michou et al., 2014; Stegemöller et al., 2017; Tawadros et al., 2012). Rater blinding to the important detail of the time point when data were collected relative to the intervention was employed in 15 studies (Argolo et al., 2013; Athukorala et al., 2014; Baijens et al., 2013; Ciucci et al., 2008; El Sharkawi et al., 2002; Hirano et al., 2015; Hunter et al., 1997; Kondo et al., 2017; Lengerer et al., 2012; Miles et al., 2017; Pitts et al., 2009; Sundstedt, Holmén, et al., 2017; Sundstedt et al., 2012; Warnecke et al., 2016).

Additional information regarding protocols across the 17 studies that used VFSS as an outcome measure is summarized in Table 3. Of these, two studies did not describe patient positioning/view (Hirano et al., 2015; Pitts et al., 2009), 11 performed the study in lateral view only (Argolo et al., 2013; Baijens et al., 2013; Bushmann et al., 1989; Khedr et al., 2019; Ciucci et al., 2008; El Sharkawi et al., 2002; Hunter et al., 1997; Lengerer et al., 2012; Michou et al., 2014; Monte et al., 2005; Troche et al., 2010), and four performed the study in both lateral and anterior–posterior views (Fuh et al., 1997; Miles et al., 2017; Tison et al., 1996; Xie et al., 2015). Seven studies did not report the videofluoroscopy frame rate. Where frame rate was reported, a single study performed VFSS at 15 frames per second (fps; Lengerer et al., 2012), two studies performed VFSS at 25 fps (Baijens et al., 2013; Hunter et al., 1997), and seven studies performed VFSS at 30 fps (Ciucci et al., 2008; Fuh et al., 1997; Michou et al., 2014; Miles et al., 2017; Monte et al., 2005; Troche et al., 2010; Xie et al., 2015). A summary of the protocols used for FEES and EMG is available in Tables 4 and 5, respectively.

**Table 3.** Additional details regarding videofluoroscopy protocols used in the selected studies.

Study	Intervention	Position	Equipment	Protocol	Analysis/blinding	Frames per second	Consistency/volume/barium
Argolo et al. (2013)	Swallowing exercises	Lateral	—	Thin liquid, thick liquid, puree, and soft solids.	Randomized and analyzed frame by frame by SLP blinded to the time point of measurement (pre- vs. posttherapy).	—	Thin liquid: spoon with 5 and 10 ml and a cup with 20 ml of thin liquid (barium mixed with water at a 1:1 ratio) Thick liquid: spoon with 5 and 10 ml and a cup with 20 ml of thick liquid (pure barium) Puree: spoon with 5, 10, and 15 ml of puree (barium mixed with Nestlé natural yogurt at a 2:1 ratio) Soft solid foods: 1/2 wafer (dipped in barium)
Baijens et al. (2013)	Neuromuscular electrical stimulation	Lateral	Philips Diagnost 97 system and a Panasonic AG-DVC30 mini-DV camcorder	Low-density barium (40% [wt/vol]), thickened barium, and crackers coated with barium paste.	Randomized. SLPs blinded to group, to time point of measurement (pre- vs. posttherapy), and to each other's ratings.	25	Three trials of 10-ml low-density barium (40% [wt/vol]) Three trials of 10-ml thickened barium (50-ml applesauce, 150-g barium powder) Three bite-sized crackers coated with barium paste
Bushmann et al. (1989)	Levodopa and carbidopa	Lateral	—	Thin liquid, thick liquid, custard, cookie, and usual medications. After baseline VFSS, patients took usual dose of levodopa. Second VFSS repeated after 90 min or subjective response. Non-PD participants only had single VFSS.	Independently rated by 2 SLPs, one of whom was blind to diagnosis.	—	Thin: 3, 5, and 10 ml Thick liquid: 3 and 5 ml Custard: 3 and 5 ml Solid: cookie Usual medications
Ciucci et al. (2008)	Deep brain stimulation	Lateral	Philips Universal R/F EasyDiagnost Eleva and Regis program	Single time point ≥ 3 months after surgery. VFSS with DBS-On and DBS-Off. 1 hr between conditions. Counterbalanced order. Standard clinical procedures were used. Instruction: “Swallow as you would typically.”	—	30	Thin: three trials each of 5 and 10 ml of water (mixed with E-Z-PAQUE barium sulfate suspension in 25:75 water-to-barium ratio) Solid: 7 g of graham cracker coated with E-Z-PASTE esophageal cream
El Sharkawi et al. (2002)	Lee Silverman Voice Treatment	Lateral	VHS video recorder	VFSS before and after 1 month of the LSVT using a standard protocol.	Clinician was blinded to the time point of measurement (pre- vs. posttherapy).	—	Thin: two each of 1, 3, 5, and 10 ml and cup-drinking of barium liquid Pudding: 2 ml of barium pudding (paste) Solid: two pieces (1/4 each) of a Lorna Doone cookies coated with barium
Fuh et al. (1997)	Levodopa	Lateral + frontal position	Super VHS tape recorder	After a baseline VFSS examination, patients took 200 mg of levodopa (in combination with 50 mg of benserazide). A second VFSS examination was begun 60–90 min later.	Rated by one observer who was blinded to symptom severity but not to the time the drugs were taken.	30	Thin: 3, 5, and 7 ml Barium paste: 3, 5, and 7 ml Cookie: 1 ml
Hirano et al. (2015)	Rotigotine transdermal patch	—	—	Screen with diluted solution of barium × 2. If swallowing was not severely impaired, concentrated solution of barium × 1 (unrestricted volume). Barium mixed with jelly was then swallowed.	One SLP and one neurologist who were blinded to all clinical details. Rating according to a scale established by the Japanese Society of Dysphagia Rehabilitation and the DOSS.	—	Diluted barium solution (5 ml) Concentrated barium Jelly (6 g) mixed with barium
Hunter et al. (1997)	Levodopa and apomorphine	Lateral	Shimadzu image intensifier and a Panasonic Super VHS recorder	VFSS performed according to a standard protocol.	Evaluated independently by two SLPs blinded to the patient and timing of the swallow in relation to the dopaminergic challenge.	25	Thin: 5 ml Semisolid: 3-ml jelly Solid: dry toast about 5.8 cm^3^
Khedr et al. (2019)	Repetitive transcranial magnetic stimulation	Lateral	GE Prestige II	VFSS was performed before and after rTMS sessions while patients were on levodopa therapy. Cued swallows.	—	—	Thin: 5 ml via spoon Semisolid: 5 ml via spoon Solid: 5 ml via spoon Cocoa added to improve flavor.
Lengerer et al. (2012)	Deep brain stimulation	Lateral	Siemens Polystar X-ray machine	Three different consistencies across three conditions (preoperative, postoperative DBS-On, and postoperative DBS-Off). Participants took usual dopaminergic medication. Mean of 20.3 months and an *SD* of 8.6 between the pre- and postoperative exams. About 10 min between the postoperative conditions (DBS-On and DBS-Off).	VFSS images were blindly rated under the supervision of an experienced linguist.	15	Viscous: 5 ml of jello Fluid: 10 ml of water Solid: bread of the size of a 2 euro coin mixed with iodine (Ultravist 240)
Michou et al. (2014)	Levodopa	Lateral	Siemens Fluorospot Compact imaging system, Siemens Sireskop SX X-ray unit, and a Videomed DI-TV system (Sony DHR-1000)	Baseline VFSS, then usual first levodopa dose. After an hour of rest, pharyngeal catheter inserted. Cortical and cranial nerve stimulation administered. Catheter removed, and a second VFSS was performed.	SLP blinded to time point and medication status.	30/25	Thin: 6 swallows of 5-mL thin liquid barium (60% [wt/vol], E-Z-HD)
Miles et al. (2017)	Lee Silverman Voice Treatment	Lateral and anterior–posterior	Toshiba DF-323H videofluoroscope and Horita VS-50 Video Stopwatch	Lateral view: thin liquid barium: 20 ml, then 100 ml by straw. Instruction: “Drink the whole cup in your own time but without stopping.” Then, 5-ml barium paste. AP view: 20-ml thin bolus. Instruction: “Drink all in one go.” If residue seen, participant asked to perform a dry swallow.	Authors blinded to participant and time point.	30	Thin: 20 ml of thin liquid barium (E-Z-PAQUE 96% [wt/vol] diluted to 19%), followed by 100 ml of thin liquid barium through a straw Barium paste: 5 ml of barium paste (E-Z-PASTE 60% [wt/wt])
Monte et al. (2005)	Levodopa	Lateral	Super VHS tape recorder	1. Thin barium × 2. Instruction to swallow all the bolus volume at once. 2. Bread × 2. Tap water rinses. On-drug, between 1 and 2 hr after last dose of levodopa.	Performed by an examiner blinded to patient identity.	30	Thin: 10 ml of thin barium suspension Solid: piece of bread 8.0 cm^3^ Tap water rinses between boluses
Pitts et al. (2009)	Expiratory muscle strength training	—	Kay Elemetrics Digital Swallowing Workstation (Model 7200)	30-m thin bolus, swallowed in a continuous manner.	SLP blinded to experimental condition.	—	Thin: 30 ml (Varibar; E-Z-EM)

*Note.* Em dashes indicate data not reported. SLP(s) = speech-language pathologist(s); VFSS = videofluoroscopic swallowing study; PD = Parkinson disease; DBS = deep brain stimulation; VHS = Video Home System; LSVT = Lee Silverman Voice Treatment; DOSS = Dysphagia Outcome and Severity Scale; rTMS = repetitive transcranial magnetic stimulation; AP = anterior–posterior.

**Table 4.** Additional details regarding fiberoptic endoscopic evaluation of swallowing (FEES) protocols used in selected studies.

Study	Intervention	Equipment	Protocol	Analysis/blinding	Consistency
Baijens et al. (2013)	VitalStim	PENTAX FNL-10RP3, Alphatron Stroboview ACLS camera, Alphatron light source, IVACX computerized video archiving system; recorded on a DVD	10-ml thin liquid × 3, 10-ml thick liquid × 3, bite-sized crackers × 3	Judges blinded to group, to time point of measurement (pre- vs. posttherapy), and to each other's ratings.	Thin liquid: water dyed with 5% methylene blue Thick liquid: applesauce dyed with 5% methylene blue
Kondo et al. (2017)	Aural stimulation with capsaicin ointment to the external auditory canal	PENTAX VNL-100S endoscope (3.1 mm in diameter)	Standard FEES protocol of The Oto-Rhino-Laryngological Society of Japan. Tested 5, 30, and 60 min after a single application of 0.5 g of 0.025% capsaicin or placebo ointment to the right external auditory canal.	Video images evaluated using endoscopic swallowing scoring and the SMRC scale by a second otolaryngologist blinded to clinical data and original ratings.	Water (3 ml) dyed with blue food coloring
Kulneff et al. (2013)	Deep brain stimulation	Olympus ENF-P4 transnasal flexible endoscope and a Wolf 5502 endocam	One solid and four different liquid consistencies. Started with thin liquid, then thicker and solid consistencies, and finished with water.	—	Thin liquid: 5 ml of jellification powder in 500 ml of water Semi-viscous liquid: 10 ml of powder in 500 ml of water Viscous liquid: 15 ml of powder in 500 ml of water Biscuit with a smear of the thickest liquid consistency on top 10 ml of water
Sundstedt et al. (2012)	Deep brain stimulation	Olympus ENF-P4 transnasal flexible endoscope and a Wolf 5502 endocam	One solid and four different liquid consistencies. Started with thin liquid, then thicker and solid consistencies, and finished with water.	Video recordings were de-identified and randomly ordered. Scored according to a predefined protocol.	Thin liquid: 5 ml of jellification powder in 500 ml of water Semi-viscous liquid: 10 ml of powder in 500 ml of water Viscous liquid: 15 ml of powder in 500 ml of water Biscuit with a smear of the thickest liquid consistency on top 10 ml of water
Sundstedt, Holmén, et al. (2017)	Deep brain stimulation	Olympus ENF-P4 transnasal flexible endoscope and a Wolf 5502 endocam. In later examinations, an Olympus ENF-VH flexible video endoscope and an Olympus CV-170 light source system.	One solid and four different liquid consistencies. For the paper, only the final 2 consistencies were analyzed.	Raters blinded to patient status, time point, DBS status, and swallowing function.	Thin: green-dyed water Solid: biscuit with a smear of green jelly
Warnecke et al. (2016)	Oral levodopa administration	Olympus ENF-P4 flexible fiberoptic rhinolaryngoscope (3.1 mm in diameter), a Storz endovision telecam SL PAL 20212020 light source, a Storz endovision telecam SL PAL 20212030 camera, a Sony DVM 14M2MDE color monitor, and a Sony SVO9500MDP video recorder	Evaluation in the off-drug condition Single oral dose of liquid levodopa Second FEES examination approximately 30–60 min after levodopa challenge Each evaluation included nine consecutive standardized test boluses.	Independently scoring in random order by two raters, blinded to patient and assessment conditions.	Pudding: three trials × 8 ml of green jelly Liquid: three trials × 5 ml of blue-dyed liquid Solid: white bread approximately 3 × 3 × 0.5 cm.

*Note.* The em dash indicates data not reported. SMRC = sensory, motion, reflex, clearance; DBS = deep brain stimulation.

**Table 5.** Additional details regarding electromyography (EMG) protocols used in selected studies.

Author, year	Intervention	EMG protocol	Equipment	Consistencies
Alfonsi et al. (2017)	Botox	Three-channel recording: (a) suprahyoid/submental muscles (sEMG), (b) cricopharyngeus muscle (needle EMG), and (c) piezoelectric transducer signal collected on neck surface over cricothyroid membrane. Water (3 ml) administered via syringe and swallowed.		
Athukorala et al. (2014)	Skill training therapy	Submental sEMG. Five saliva and five 10-mL water swallows with task types randomized within and between participants. Instructions to, “Hold the water/ saliva in your mouth and when you hear the go signal, swallow as quickly as possible.” Average premotor time, preswallow time, duration of submental muscle contraction calculated for each task, at each session, per participant.	KayPENTAX Digital Swallowing Workstation	
Stegemöller et al. (2017)	Singing	Right and left submental and laryngeal sEMG. Amplitude and timing measures. Three swallows each for thin and thick stimuli. EMG amplitudes were not normalized.	Delsys Trigno EMG sensors, The Motion Monitor soft-ware (Innovative Sports Training, Inc.)	Thin: 10 ml of water; Thick: 10 ml of pudding
Tawadros et al. (2012)	Levodopa	Data collected in morning in off-levodopa state and repeated 1 hr after self-administration of regular morning medication. Submental sEMG was filtered and laryngeal accelerometry signals collected. Baseline measurements made during rest. A 9-s postswallow clearing phase also measured. EMG parameters included peak amplitude, burst area and duration, and rise time and fall time. Time between onset of submental and laryngeal burst was also collected. Duration and peak amplitude of accelerometry signals calculated.	2g piezo-electric accelerometer (IC Sensors model 3145), Grass^TM^ 15LT Astro-Med, Inc, National Instruments™ BNC-2120, LabVIEW 7	Six water boluses (3, 5, 10, 15, 20, and 25 ml). Three repetitions of each. A subset of participants also drank a 100-ml “stress test” bolus.

EMG = electromyography; sEMG = surface electromyography; N/A = not applicable.

## Discussion

### Summary of Findings

Of the 26 studies included for synthesis, 11 described pharmacological interventions, eight investigated the effects of neurostimulation, and seven described outcomes of behavioral interventions. Although several studies concluded that posttreatment improvements were seen in swallowing safety, efficiency, and timing measures, findings were inconsistent, and the quality of the evidence was generally low based on high RoB and low instrumental rigor ratings. Notwithstanding these concerns, overall, low certainty evidence across more than one study suggested the following trends:

1. DBS and exercises guided using sEMG biofeedback may lead to improvements in swallow timing/latency (Athukorala et al., 2014; Ciucci et al., 2008; Lengerer et al., 2012).
2. EMST and DBS may lead to improvements in swallowing safety (Pitts et al., 2009; Troche et al., 2010; Xie et al., 2015).
3. The LSVT and pharmacological intervention with levodopa may lead to improvements in swallowing efficiency (Bushmann et al., 1989; El Sharkawi et al., 2002; Michou et al., 2014; Miles et al., 2017; Warnecke et al., 2016).

As a group, those studies rated to have a lower RoB and greater experimental rigor reported improvements in swallowing efficiency but little effect on swallowing safety (Hunter et al., 1997; Khedr et al., 2019; Kulneff et al., 2013; Michou et al., 2014; Miles et al., 2017; Sundstedt, Holmén, et al., 2017; Sundstedt et al., 2012; Troche et al., 2010). However, mild baseline impairment was identified to be a common limitation (Lengerer et al., 2012; Miles et al., 2017; Troche et al., 2010) and may have introduced a ceiling effect that obscured signs of improvement. Additionally, given that inadequate reporting of methodological details about contrast media, signal acquisition settings, and rater blinding to time point was identified as a concern in the majority of the selected studies, these apparent trends in results should be interpreted with caution. Pooling of data across studies was determined to be inappropriate given the significant heterogeneity seen across studies in videofluoroscopy, endoscopy, and EMG protocols and methods of measurement.

### Limitations Associated With Instrumental Rigor

In addition to mild baseline impairment limiting generalizability, the appraisal of rigor in instrumental evaluations revealed several other concerns regarding the selected studies. A number of studies included participants with significant differences in baseline dysphagia and PD severities within groups, with no subgroup analyses to distinguish outcomes based on severity. The generalizability of outcomes to individuals with both mild and severe baseline impairment in these cases is questionable. Additionally, several studies demonstrated limitations in their instrumental protocols when evaluating effectiveness of treatments by basing their conclusions on trials involving single fluid consistencies (Alfonsi et al., 2017; Kondo et al., 2017; Pitts et al., 2009; Tawadros et al., 2012; Tison et al., 1996; Troche et al., 2010) and single boluses (Alfonsi et al., 2017; Kondo et al., 2017; Pitts et al., 2009; Tison et al., 1996). The results of these studies must therefore be interpreted with caution, given that findings may not be replicable, at both within-participant and across-participants levels. Furthermore, of the 20 studies with multiple assessors, 10 failed to explicitly report on interrater reliability (Alfonsi et al., 2017; El Sharkawi et al., 2002; Hirano et al., 2015; Khedr et al., 2019; Kondo et al., 2017; Michou et al., 2014; Stegemöller et al., 2017; Tawadros et al., 2012; Tison et al., 1996; Xie et al., 2015). Given the subjectivity associated with rating instrumental outcomes, such reliability is important to ensure reproducibility and accuracy of findings.

### Findings in Context

Our findings are concordant with and build on those of another recent systematic review by López-Liria et al. (2020). Both reviews emphasize the lack of substantial scientific evidence comparing the effectiveness of the various techniques described, highlighting that more work needs to be done to establish or define what types of rehabilitation techniques, maneuvers, and exercises are effective for dysphagia management in PD. However, our systematic review goes beyond this to highlight important limitations with regard to the methodological rigor and RoB in the included studies and emphasizes that findings must be interpreted in the context of overall low certainty evidence. In the López-Liria et al. review, RoB was evaluated using the Jadad scale (Jadad et al., 1996). According to this scale, studies meeting three or more of the following criteria are rated as having a low RoB: (a) The study is described as randomized, (b) an appropriate method of generating the randomization sequence is described, (c) the study employed and describes appropriate blinding, and (d) participant dropouts and loss to follow-up are fully reported. Using these criteria, López-Liria et al. found only one study to be of low quality. Our results are discordant with this appraisal, identifying serious RoB and poor experimental rigor as concerns in the majority of studies reviewed. Using the Evidence Project tool, which is specifically designed to capture RoB across a range of study designs, our review highlights additional gaps in methodological rigor related to the inclusion of control/comparison groups, the equivalence of groups on sociodemographics and that at baseline, and random selection of participants from eligible pools. In addition, our review involves further scrutiny of study for important rigor and transparency criteria pertinent to dysphagia clinical practice, particularly with respect to descriptions of the instrumental examination protocols used for measuring outcomes. Evaluation of these additional domains provides a further nuanced appraisal of the effectiveness of these interventions. By emphasizing these limitations, our review encourages end-user clinicians to interpret findings with caution and to critically appraise the interventions implemented in their clinical practice. In addition, these limitations may shed light on the reasons for significant practice variation.

Previous efforts to synthesize evidence regarding the effectiveness of pharmacological, neurostimulation, and behavioral interventions for dysphagia in PD have yielded limited results or have limited their scope to specific intervention approaches, study designs, search periods, and/or databases (Ashford et al., 2009; Baijens et al., 2009; Battel et al., 2021; Chang et al., 2021; Deane et al., 2001; Grimes et al., 2019; Smith et al., 2012; Van Hooren et al., 2014). Conclusions from these systematic reviews highlight the lack of sufficient evidence to support or refute swallowing therapies in PD due to a limited number of well-designed studies (Baijens et al., 2009; Chang et al., 2021; Deane et al., 2001), with some attributing the inconsistency of results across previous studies to the inclusion of different stages of PD and the use of different evaluation tools for dysphagia in each study (Chang et al., 2021). Most of these reviews concluded that further investigations are warranted, including large, randomized sham-controlled trials (Chang et al., 2021; Van Hooren et al., 2014).

This systematic review represents an effort to synthesize and compare evidence in a comprehensive manner across three types of intervention, utilizing a broad search strategy and no limits in terms of study design and date of publication. Overall, our findings concur with the findings of previous reviews, suggesting very low certainty evidence to guide practice.

Currently, there is a lack of formal guidance around treatment for oropharyngeal dysphagia in people with PD in North American professional practice guidelines. Guidelines from other countries provide weak recommendations for EMST (Grimes et al., 2019) and the LSVT based on very low certainty evidence (Kalf et al., 2011; Ministry of Health, Social Services, and Equality & Institute of Health Sciences of Aragon, 2014). Beyond this, current guidelines provide little direction regarding treatment choices for dysphagia in PD. This reflects a situation of clinical equipoise as well as a limited and low certainty evidence base, which precludes our ability to make recommendations to guide clinical practice (Kamal et al., 2012).

### Limitations of the Review

Our review had several limitations. First, instrumental outcomes were limited to VFSS, FEES, and EMG; while outcomes evaluated by manometry and other instrumental techniques may provide additional data regarding PD-related dysphagia interventions, we chose the three most commonly used instrumental measures. Second, only studies with quantitative measures of effect from before–after or parallel-arm comparisons were included; qualitative descriptions of effect or single-arm studies were excluded, but these may provide additional insight into the benefits and harms of available interventions. Third, we found that the Evidence Project tool does not capture some elements of quality that other tools assess. For example, the Cochrane RoB tool (Higgins et al., 2019) includes blinding of participants and personnel, blinding of outcome assessment, and selective reporting, all of which are key domains in RoB assessment. However, given that not all included studies were RCTs, this tool was not used. Additionally, the lack of a numeric RoB summary score representing the overall quality across included articles poses a challenge to succinctly summarizing the overall RoB in the review. Finally, interventions targeting cough strength, respiratory function, and overall physiologic reserve were beyond the scope of this review article.

## Conclusions

Future research is needed to elucidate the effects of pharmacological, neurostimulation, and behavioral interventions for dysphagia in PD by implementing standard protocols targeting specific physiological mechanisms related to swallowing safety, efficiency, and timing and rigorous measurement of outcomes using videofluoroscopy, endoscopy, EMG, or other tools. Specifically, there is a need to design future studies with the following considerations:

1. Given the lack of clear evidence that the aforementioned interventions impact the frequency of penetration–aspiration, studies should expand their focus to measuring changes in other physiological parameters related to airway protection. This could be done by studying parameters that capture the integrity of laryngeal vestibule closure and the time needed to achieve laryngeal vestibule closure (Curtis et al., 2020a, 2020b).
2. Future studies investigating improvements in swallowing efficiency and timing as outcomes should measure parameters related to pharyngeal constriction, pharyngeal shortening, and UES opening (Curtis et al., 2020a, 2020b).
3. Future studies need to provide much greater detail regarding the methods used to collect and interpret instrumental measures of swallowing. Thorough descriptions of methods that permit replication and provide evidence of experimental rigor are needed. In particular, protocols should include trials across various consistencies of food and fluids, a sufficient number of swallowing tasks to account for variability within a person, details about the type and concentration of barium used, and equipment used for data collection to be specified for replicability purposes.
4. In order to provide strong evidence of treatment effect, studies should strive to compare study groups that are equivalent on sociodemographic factors and baseline swallowing function, with confirmation that baseline function is unequivocally impaired on the parameters of interest.

Robust studies with due consideration to these elements are warranted to guide evidence-based clinical practice.

## Author Contributions

**Pooja Gandhi:** Conceptualization (Equal), Methodology (Lead), Investigation (Equal), Formal analysis (Lead), Validation (Equal), Writing - original draft (Lead), Writing - review & editing (Equal). **Catriona M. Steele:** Conceptualization (Equal), Investigation (Equal), Data curation (Lead), Validation (Equal), Writing - review & editing (Equal).

## Supplementary Material

10.1044/2021_AJSLP-21-00145SMS1 Supplemental Material S1 Summary of study characteristics.

## Appendix

Full Details of the Search Strategy Used in This Review Article

**Ovid MEDLINE ALL <1946 to May 20, 2019>**

**Search history sorted by search number ascending**

**Table:**

No.	Searches	Results
1	exp Parkinsonian Disorders	74,758
2	Parkinson Disease	61,685
3	parkinson*.tw,kf,jn.	109,844
4	(lewy adj2 bod*).tw,kf.	8,623
5	paralysis agitans.tw,kf.	1,172
6	1 or 2 or 3 or 4 or 5	121,588
7	exp Deglutition Disorders	50,198
8	Deglutition	9,223
9	dysphag*.tw,kf,jn.	26,666
10	deglut*.tw,kf.	4,407
11	swallow*.tw,kf.	27,696
12	7 or 8 or 9 or 10 or 11	84,845
13	6 and 12	1,064
14	13 not (exp animals/ not exp humans/)	1,049
15	14 not ((exp infants/ or exp children/) not exp adults/)	1,039
16	limit 15 to english language	888

**Cochrane Central Register of Controlled Trials <2014 to Present>**

**Search history sorted by search number ascending**

**Table:**

No.	Searches	Results
1	exp Parkinsonian Disorders	3,805
2	Parkinson Disease	3,621
3	parkinson*.tw,kf,jn.	9,069
4	(lewy adj2 bod*).tw,kf.	354
5	paralysis agitans.tw,kf.	10
6	1 or 2 or 3 or 4 or 5	9,442
7	exp Deglutition Disorders	2,559
8	Deglutition	352
9	dysphag*.tw,kf,jn.	3,239
10	deglut*.tw,kf.	127
11	swallow*.tw,kf.	4,129
12	7 or 8 or 9 or 10 or 11	8,085

**Embase Classic + Embase <1947 to May 20, 2019>**

**Table:**

No.	Searches	Results
1	parkinsonism	30,678
2	Parkinson disease	143,921
3	parkinson*.tw,kw,jn.	162,520
4	(lewy adj2 bod*).tw,kw.	13,453
5	paralysis agitans.tw,kw.	494
6	1 or 2 or 3 or 4 or 5	200,869
7	dysphagia	66,007
8	swallowing	23,295
9	dysphag*.tw,kw,jn.	47,989
10	deglut*.tw,kw.	6,600
11	swallow*.tw,kw.	45,782
12	7 or 8 or 9 or 10 or 11	109,644
13	6 and 12	3,116
14	13 not ((exp animals/ or exp animal experimentation/ or nonhuman/) not exp human/)	3,044
15	14 not ((exp embryo/ or exp fetus/ or exp juvenile/) not exp adult/)	2,979
16	limit 15 to (conference abstract or conference paper or “conference review”)	1,010
17	15 not 16	1,969
18	17 not medline.cr.	1,799
19	limit 18 to english language	1,622

**CINAHL With Full Text (May 21, 2019)**

**Table:**

No.	Query	Limiters/expanders	Results
S1	(MH “Parkinsonian Disorders+”)	Search modes – Boolean/Phrase	19,196
S2	(MH “Parkinson Disease”)	Search modes – Boolean/Phrase	17,952
S3	TI parkinson* OR AB parkinson* OR SO parkinson*	Search modes – Boolean/Phrase	23,479
S4	AB (lewy n2 bod*) OR TI (lewy n2 bod*)	Search modes – Boolean/Phrase	1,768
S5	TI paralysis agitans OR AB paralysis agitans	Search modes – Boolean/Phrase	10
S6	S1 OR S2 OR S3 OR S4 OR S5	Search modes – Boolean/Phrase	27,457
S7	(MH “Deglutition Disorders”)	Search modes – Boolean/Phrase	7,139
S8	(MH “Deglutition”)	Search modes – Boolean/Phrase	3,196
S9	TI dysphag* OR AB dysphag* OR SO dysphag*	Search modes – Boolean/Phrase	7,915
S10	TI deglut* OR AB deglut*	Search modes – Boolean/Phrase	388
S11	TI swallow* OR AB swallow*	Search modes – Boolean/Phrase	7,506
S12	S7 OR S8 OR S9 OR S10 OR S11	Search modes – Boolean/Phrase	15,320
S13	S6 AND S12	Search modes – Boolean/Phrase	416
S14	S13	Limiters – English Language search modes – Boolean/Phrase	405

**speechBITE – Advanced Search (May 22, 2019)**

**Table:**

No.	Searches	Results
1	Keyword: Parkinson* SLP Practice Area: Swallowing	41
2	Keyword: Parkinson* SLP Practice Area: Dysphagia	35 (no new articles added)
3	Keyword: Parkinson* Dysphagia	36 (1 new article added)
