# Supplementary material for: Effectiveness of Interventions for Dysphagia in Parkinson Disease: A Systematic Review
Source: Am J Speech Lang Pathol. 2021 Dec 10;31(1):463–85. doi: 10.1044/2021_AJSLP-21-00145 (PMC9159671; doi:10.1044/2021_AJSLP-21-00145)
Supplement: Supplemental Material S1 [file AJSLP-31-463-s001.pdf]

Supplemental Material S1. Summary of study characteristics.

Author, Year	Type of Intervention	Study Design	Result Summary	Primary Outcome Domain(s)	Improvements?
Alfonsi et al. 2017	Botox to UES	B-A	• 10/12 showed improvements after an initial injection.	UES opening	Yes
Argolo et al., 2013	Oral motor exercises	B-A	• Groups were not equivalent at baseline on lingual pumping, teeth absence and dyskinesia.<br>• Lingual pumping and dental absence were confounding factors associated with treatment failure.<br>• Transit time measures were not reduced after the intervention.	Timing/Latency	No
Athukorala et al., 2014	EMG Guided Skill training therapy	B-A	• Dry swallows: significant effect of time for premotor time, preswallow time, duration of submental muscle contraction.<br>• No effect on duration of submental muscle contraction for water swallows.	Timing/Latency	Yes
Baijens et al., 2013	Neuromuscular electrical stimulation	RCT	• No differences for any visuoperceptual FEES and VFSS variables.	Various	No
Bushmann et al., 1989	Levodopa (with Carbidopa)	RCT	• Five patients with abnormal swallows improved after Levodopa.<br>• The most frequently observed improvement was decreased vallecular residue and 8/12 medication, with one patient’s swallow worsening.<br>• Increased doses of Levodopa (100 to 125 mg) did not improve swallowing.	Efficiency, pharyngeal transit	Yes, partially
Ciucci et al., 2008	Deep brain stimulation	B-A	• Longer pharyngeal transit times occurred in the off condition. No differences in hyoid excursion and oral total composite score.<br>• Pharyngeal stage impairment was worse in the off condition.	Timing/Latency and Pharyngeal Composite Score	Yes
El Sharkawi	Lee Silverman	B-A	• Reduction in the number of swallowing	Efficiency/residue	Yes

et al., 2002	Voice Treatment		motility disorders.<br>• Some timing measures were significantly reduced post treatment.<br>• Reduced oral residue for 3 ml and 5 ml liquid swallows post treatment. Improvements in oral tongue and tongue base as well as improved vocal intensity.		
Fuh et al., 1997	Levodopa (with Benserazide)	RCT	• Of 12 patients with abnormal swallows, 6 showed improvement. One patient showed oral phase improvement but a worse pharyngeal phase.<br>• Of 6 patients with oral phase impairment, 2 showed improvements in oral tremor and 1 in tongue elevation.<br>• Of 3 patients with aspiration, 2 showed no post-Levodopa aspiration.<br>• Graphic representation of pharyngeal phase abnormalities suggested that vallecular and pyriform sinus residue improved post-Levodopa.	Various parameters	Mixed
Hirano et al., 2015	Rotigotine Patch	RCoh	• Oral and pharyngeal phase improvement in all six patients (pharyngeal transit duration was significantly shortened).	Efficiency, pharyngeal transit	Yes
Hunter et al., 1997	Levodopa (with Apomorphine)	RCT	• Shorter oral preparatory phase with both semisolids and thin fluids. Longer oral phase and total initial swallow durations with solids. Shorter rapid pharyngeal transit time with semisolids after apomorphine.	Timing/Latency and Efficiency	Yes
Khedr et al., 2019	Repetitive transcranial magnetic stimulation (rTMS)	RCT	• No difference between sham or real rTMS groups in penetration-aspiration (PAS) or residue.<br>• Significant increase in maximum hyoid elevation and decrease in pharyngeal transit time for fluids	Safety, Efficiency, Timing	No
Kondo et al., 2017	Aural stimulation, capsaicin	RCT	• Endoscopic swallowing scores of treatment group were significantly decreased 30 and	Glottal closure, timing, efficiency	Yes

	ointment		60 minutes after single aural administration of capsaicin ointment. 1/10 participants in the experimental group had a diagnosis of PD. The placebo group, including 2 patients with PD, showed no change.		
Kulneff et al., 2013	Deep brain stimulation	PCoh	• No significant effect of the intervention on FEES parameters including PAS, Secretion Scale, pharyngeal residue, pre-swallow spillage, and bolus clearance.	Safety, Residue, Spillage	No
Lengerer et al., 2012	Deep brain stimulation	RCoh	• Preoperatively, none of the patients presented with clinically relevant signs of dysphagia.<br>• Postoperatively, mean daily levodopa equivalent dosage was reduced.<br>• Significant changes in pharyngeal parameters with deep-brain-stimulation-on compared to preoperative condition and deep-brain stimulation off.	Timing/Latency	Yes
Michou et al., 2014	Levodopa	B-A	• While on Levodopa, bilateral increases in pharyngeal cortical excitability were noted compared to those with no swallowing impairment. Amplitudes of brainstem reflexes decreased in patients with swallowing impairment in the on-Levodopa condition compared to the off-drug state.	Safety, Latency	Yes, partially
Miles et al., 2017	Lee Silverman Voice Treatment	PCoh	• No episodes of PAS (constant score of 1).<br>• Pharyngeal residue and pharyngeal area at rest reduced.<br>• Significantly increased duration and maximal opening of pharyngo-esophageal segment.<br>• Significant improvements in involuntary cough, peak expiratory flow rate and rise time.	Efficiency, UES Opening Diameter	Yes
Monte et al., 2005	Levodopa	RCT	• No significant difference between non-dyskinetic patients, dyskinetic, and controls in oral and pharyngeal transit times.<br>• Non-dyskinetic patients had more	Efficiency	No

			pharyngeal retention of liquid and solid food and worse swallowing efficiency than controls.		
Pitts et al., 2009	Expiratory Muscle Strength Training	B-A	• Significant reduction in PAS after training.	Safety	Yes
Stegemöller et al., 2017	Therapeutic singing	PCoh	• Laryngeal muscle group surface EMG: No changes in area under the curve or peak amplitude for swallows of thin and thick liquids. Submental muscle group surface. EMG: No effects.	Timing/Latency	No
Sundstedt et al., 2012	Deep Brain Stimulation	PCoh	• Pre- and post-operative comparisons revealed a significant reduction of pre-swallow spillage at 6 months in both on and off stimulation condition.<br>• No differences between stimulation on and off conditions 6-months post.<br>• Significantly more pre-swallow spillage in the stimulation on condition versus off condition 12 months post.	Safety, Residue, Spillage	No
Sundstedt et al., 2017	Deep Brain Stimulation	B-A	• No significant changes in PAS, pharyngeal residue or premature spillage.	Safety, Residue, Spillage	No
Tawadros et al., 2012	Levodopa	RCT	• Larger boluses produced higher submental EMG burst amplitudes, longer durations, larger areas and longer rise and fall times across both groups.<br>• No significant effect of levodopa on any submental burst parameters, laryngeal parameters or number of swallows at any volume.	Various surface EMG parameters	No
Tison et al., 1996	Apomorphine (with Domperidone)	RCT	• Vallecular stasis (7/8 patients) was improved by apomorphine in 4 cases. Fragmentation of the bolus (7/8 patients) was improved by apomorphine in 3 cases. Buccal stagnation of the bolus improved in 3 cases. Two of 3 patients with laryngeal penetration, showed improvement after	Various parameters	Yes

			apomorphine.		
Troche et al., 2010	Expiratory Muscle Strength Training	RCT	• Significant reductions in PAS scores in the experimental group but not in the sham control group.<br>• Significantly shorter hyoid durations in the sham group. Pattern of increased hyoid displacement at all timepoints in the EMST group but decreased hyoid displacement at all timepoints in sham group.	Safety, kinematics of hyoid movement	Yes
Warnecke et al., 2016	Levodopa	B-A	• 7 patients showed improvement of dysphagia in the on-drug state. 8 patients did not respond. Improvements in premature spillage and residue for semisolid and solid consistencies.	Residue, spillage	Yes
Xie et al., 2015	Deep brain stimulation	RCT	• Compared to 130 Hz, 60-Hz stimulation significantly reduced aspiration frequency by 57%. The benefits at 60-Hz stimulation persisted over the average 6-week assessment.	Safety	Yes

Note. VFSS = Videofluoroscopy; FEES = Fiberoptic Endoscopic Examination of Swallowing; EMG = Electromyography; PAS = Penetration-Aspiration Scale; UES = Upper Esophageal Sphincter; TMS = Transcranial Magnetic Stimulation; LSVT = Lee Silverman Voice Treatment; EMST = Expiratory Muscle Strength Training; RCT = Randomized Control Trial; B-A = Before-After Trial; PCoh = Prospective Cohort Study; RCoh = Retrospective Cohort Study.
